# High-quality draft genome sequence of a biofilm forming lignocellulolytic *Aspergillus niger* strain ATCC 10864

**DOI:** 10.1186/s40793-017-0254-2

**Published:** 2017-07-17

**Authors:** Sujay Paul, Yvette Ludeña, Gretty K. Villena, Fengan Yu, David H. Sherman, Marcel Gutiérrez-Correa

**Affiliations:** 1 Laboratorio de Micología y Biotecnología, Universidad Nacional Agraria La Molina, Av. La Molina s/n, 12 Lima, Peru; 2 Life Sciences Institute, University of Michigan, 210 Washtenaw Avenue, Ann Arbor, MI 48109-2216 USA

**Keywords:** *Aspergillus niger*, Lignocellulolytic enzyme, Biofilm, Genomic feature

## Abstract

Filamentous fungus *Aspergillus niger* has high industrial value due to their lignocellulolytic enzyme activities and ATCC 10864 is one of the few type strains of *A. niger* which has a unique biofilm forming capability. Here we report the first draft genome sequence of *A. niger* ATCC 10864 strain. The genome of *A. niger* ATCC 10864 is 36,172,237 bp long and comprise of 310 scaffolds with 49.5% average GC content. A total of 10,804 protein-coding genes were predicted among which 10,761 genes were with putative functions. *A. niger* ATCC 10864 genome coded for 709 putative carbohydrate active enzyme families distributed in six functional categories and among them glycoside hydrolases (GHs) represent the most number of families (279). Genes that include *pepA*, *brlA*, *exgA*, *LaeA*, *rodA*, *GCN* have also been identified in this study, which may play a role in biofilm formation. This high-quality draft genome sequence will facilitate our understanding of the mechanisms behind fungal biofilm formation and higher lignocellulolytic enzyme production.

**Electronic supplementary material:**

The online version of this article (doi:10.1186/s40793-017-0254-2) contains supplementary material, which is available to authorized users.

## Introduction

Filamentous fungi mostly are considered as cell factories because of their ability to produce enzymes involved in the conversion of lignocellulosic compounds to simple sugars. Among these, *Aspergillus niger*, a GRAS microorganism, is considered a model and has been used in many industrial processes [1, 2]. *A. niger* strain ATCC 10864 (CBS 122.49; IAM 2533; IAM 3009; IFO 6661; IMI 60286; JCM 22343; NBRC 6661; NRRL 330; WB 330) was previously reported to have an ability to form biofilms on polyester cloth [3–6] and interestingly, the biofilm culture of this strain can produce 50-70% more lignocellulolytic enzymes than that of conventional submerged culture [4, 7, 8]. However, due to lack of genome sequence data of this strain, the relation between biofilm formation and higher enzyme production is not well understood at the molecular level. In this context, here we illustrate a summary classification and a set of the features of *A. niger* strain ATCC 10864 with a high-quality draft genome sequence description and annotation.

## Organism information

### Classification and features

**Fig. 1:**
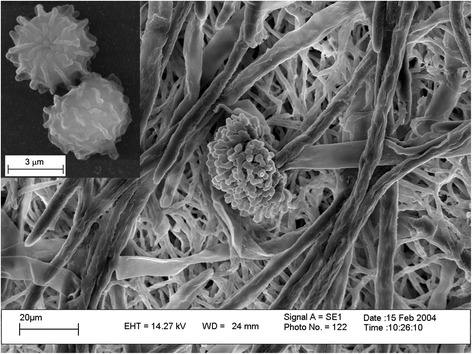
Electron micrograph of *Aspergillus niger* strain ATCC 10864. Inset: ornamented spores

**Fig. 2:**
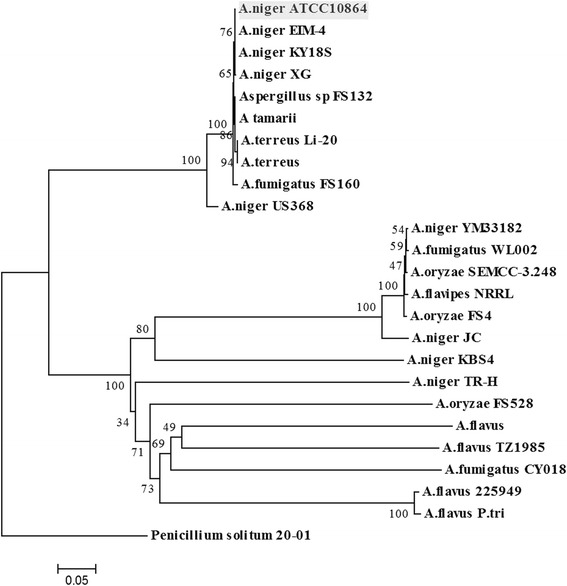
Phylogenetic tree showing the close relationship of *A. niger* strain ATCC 10864 (*grey block*) with other *A. niger* strains based on aligned sequences of the 18S rRNA gene. Multiple sequence alignment was performed using ClustalX program and the phylogeny was calculated using maximum likelihood method based on the Tamura-Nei model. The bootstrap value was inferred from 1000 replicates. *Penicillium solitum* is considered as the outgroup for this analysis. The whole analysis was carried out using MEGA5 package [43]

*Aspergillus niger* strain ATCC 10864 is a haploid, filamentous, black ornamented asexual spore (conidia) producing fungi belonging to the order *Eurotiales* and family *Trichocomaceae* (Fig. 1) and probably originated in Budapest, Hungary [9]. It is most commonly found in mesophilic environments such as decaying vegetation or soil with growth temperature from 6 °C–47 °C [10] and optimal growth at 25-35 °C [11] as well as a wide pH range: 1.4-9.8 (Table 1). Hyphae of *A. niger* ATCC 10864 are septate, hyaline and the conidiophores are long, smooth-walled, hyaline, becoming darker at the apex and ending in a globose to subglobose vesicle [12]. Phylogenetic analysis was performed by the maximum likelihood method based on 18S rRNA gene sequences and the analysis revealed close relationship of our strain with other type strains of *A. niger* (Fig. 2). Although the genomes of three *A. niger* type strains (CBS 513.88, ATCC 1015 and SH2) have already been sequenced [13–15], biofilm forming and high productive strain of *A. niger* such as ATCC 10864 is still being neglected and only very few sequence information are available in the databases.

**Table 1:** Classification and general features of *Aspergillus niger* strain ATCC 10864

MIGS ID	Property	Term	Evidence code^a^
	Current classification	Domain *Fungi*	TAS [37]
		Phylum *Ascomycota*	TAS [37]
		Class *Eurotiomycetes*	TAS [37]
		Order *Eurotiales*	TAS [37]
		Family *Trichocomaceae*	TAS [37]
		Genus *Aspergillus* (*section Nigri*)	TAS [37]
		Species *Aspergillus niger*	TAS [37]
		Strain ATCC 10864	
	Gram stain	N/A	
	Cell shape	Septate and hyaline hyphae	TAS [38]
	Motility	Non-motile	NAS
	Sporulation	Carbon black ornamented spores from biseriate phialides	TAS [39]
	Temperature range	6 °C–47 °C	TAS [10]
	Optimum temperature	25-35 °C	TAS [11]
	pH range	1.4–9.8	TAS [10]
	Carbon source	Organic carbon source	TAS [40]
MIGS-6	Habitat	In soil, litter, compost and on decaying plant material	TAS [10]
MIGS-6.3	Salinity	0-5%	TAS [41]
MIGS-22	Oxygen	Aerobic	TAS [10]
MIGS-15	Biotic relationship	Plant and animal	TAS [42]
MIGS-14	Pathogenicity	Mild/Opportunistic	TAS [10]
MIGS-4	Geographic location	Budapest, Hungary	TAS [9]
MIGS-5	Sample collection time	Unknown	
MIGS-4.1	Latitude	Unknown	
MIGS-4.2	Longitude	Unknown	
MIGS-4.3	Depth	Unknown	
MIGS-4.4	Altitude	Unknown	

^a^Evidence codes - TAS traceable author statement (i.e., a direct report exists in the literature), NAS non-traceable author statement

## Genome sequencing information

### Genome project history

Experimental studies with *A. niger* ATCC 10864 have provided four reasons to select this strain for whole genome sequencing: 1) This is the first reported biofilm forming *A. niger* strain [16]. 2) Biofilm culture of this strain can produce 2-3 times more lignocellulolytic enzymes compared to conventional submerged culture [4, 7, 8]. 3) The key mechanism that controls higher levels of enzyme production of the organism in biofilm culture is still unclear. 4) The genomes of only three strains of such an industrially relevant fungus are available in the databases [13–15]. A high-quality draft genome sequence has been deposited both in Genomes On Line Database (GOLD) [17] and DDBJ/EMBL/GenBank under accession numbers Gp0155299 and MCQH00000000 respectively. Table 2 presents the project information and its association with the minimum information about a genome sequence version 2.0 compliance [18].

**Table 2:** Project Information

MIGS ID	Property	Term
MIGS-31	Finishing quality	High-quality draft (Full genome representation)
MIGS-28	Libraries used	libraries of 400–450 bp
MIGS-29	Sequencing platforms	Illumina HiSeq 2000 (100*2)
MIGS31.2	Fold coverage	88.19 X
MIGS-30	Assemblers	SPAdes v 3.1
MIGS-32	Gene calling method (Gene prediction tool)	Augustus 3.0.3
	Locus tag	Not indicated
	Genbank ID GOLD ID	MCQH00000000 Gp0155299
	GenBank Date of Release	29-AUG-2016
	Bioproject	PRJNA300350
MIGS-13	Source material identifier	ATCC 10864
	Project relevance	Industrial

### Growth conditions and genomic DNA preparation

Duff (1988) [19] medium was used in this study to culture *A niger* strain ATCC 10864. The culture medium contained per liter: 2 g KH_2_PO_4_; 1.4 g (NH_4_)_2_SO_4_; 0.3 g urea; 0.3 g CaCl_2_.2H_2_0; 0.3 g MgSO_4_.7H_2_0; 1 g peptone; 2 ml Tween 80; 5 mg FeSO_4_.7H_2_O; 1.6 mg MnSO_4_.2H_2_O; 1.4 mg ZnSO_4_.7H_2_O; 2 mg CoCl_2_.6H_2_O; and 10 g lactose. The initial pH was set as 5.5. Thirty ml of the culture medium in 125 ml flasks was inoculated with 0.9 ml spore suspension (1 × 10^6^ spores/ml.) to each flask and incubated at 28 °C in a shaker bath (175 rpm) for 3 days. After recovery of the pellets using Whatman filter paper N° 1 (Whatman, Inc., Clifton, NJ), genomic DNA was extracted using the Wizard® Genomic DNA Purification Kit following manufacture’s instructions (Promega, Madison, WI, USA) and finally subjected to an additional purification step using the same purification kit. The quality and quantity of the purified DNA sample were evaluated by agarose gel electrophoresis and by using a NanoDrop1000 Spectrophotometer (Thermo Scientific, Wilmington, DE, USA).

### Genome sequencing and assembly

The draft genome sequence of *A. niger* ATCC 10864 was generated at the University of Michigan Life Science Institute (Michigan, USA) using Illumina technology. Genomic DNA samples were sheared to approximately 400-450 bp fragment size, then Illumina-compatible sequencing libraries were prepared from those fragments on an Apollo 324 robotic workstation (WaferGen Biosystems), using the Kapa HTP Library Preparation Kit (KAPABiosystem) according to the manufacturer’s protocols. Subsequent libraries were sequenced on an Illumina HiSeq 2000 (100*2) platform with coverage of approximately 88.19X. Approximately, 1.59 million Illumina paired-end raw reads were generated, which was quality checked using FastQC 2.2 [20] and processed for adapters and low-quality (<Q20) bases trimming. The trimmed reads were taken for further analysis.We have used in-house Perl script [21] for trimming adaptor and low-quality regions from the raw reads. Finally approx. 1.53 million reads were processed for assembly and annotation. *De-novo* assembly of Illumina paired-end data was performed using SPAdes assembler 3.1 [22] and assembled contigs were further scaffolded using SSPACE program [23]. Project information is shown in Table 2.

### Genome annotation

The resulted scaffolds were predicted for proteins using Augustus 3.0.3 [24], and subsequently annotated using NCBI BLAST 2.2.29, e-value 0.00001 [25] with the proteins of the genera *Aspergillus* taken from Uniprot database (2016_1 release) [26]. Gene ontology (GO) terms of the predicted proteins in *A. niger* strain ATCC 10864 were assigned using Blast2GO tool version 4.0.7. Secondary metabolite (SM) clusters and pathway analyses were conducted by antiSMASH 3.0 [27] and KAAS (KEGG Automatic Annotation Server) [28] tools respectively. The *A. niger* ATCC 10864 proteins were subjected for CAZymes (Carbohydrate-Active Enzymes) annotation using dbCAN (dbCAN HMMs 5.0) [29] servers, which are based on the CAZy database classification (2013 release) [30].

## Genome properties

The assembly of the draft genome sequence consists of 310 scaffolds amounting to 36,172,237 bp, and the G + C content is 49.50% (Table 3). It included a predicted 10,804 protein coding genes among which majority of genes (98.06%) assigned a putative function. Additionally, 169 (1.56%) RNA genes, 8430 (76.82%) genes with Pfam domains, 994 (9.05%) genes with signal peptides, and 2362 (21.86%) genes with transmembrane helices have also identified in this study (Table 3). Among all the predicted genes, 7509 (69.50%) were placed in 25 general COG functional gene categories. The distribution of the predicted genes, which are annotated with COG functional categories, is presented in Table 4.

**Table 3:** Genome statistics

Attribute	Value	% of Total
Genome size (bp)	36,172,237	100.0
DNA coding (bp)	17,676,693	48.87
DNA G + C (bp)	17,905,257	49.50
DNA scaffolds	310	
Total genes	10,973	100.0
Protein coding genes	10,804	98.45
RNA genes	169	1.56
Pseudo genes	Unknown	
Genes in internal clusters	Unknown	
Genes with function prediction (GO annotated proteins)	10,761	98.06
Genes assigned to COGs	7509	69.50
Genes with Pfam domains	8430	76.82
Genes with signal peptides	994	9.05
Genes with transmembrane helices	2362	21.86
CRISPR repeats	N/A

**Table 4:** Number of genes associated with general COG functional categories

Code	Value	% of total	Description
J	324	2.99	Translation, ribosomal structure and biogenesis
A	29	0.20	RNA processing and modification
K	337	3.11	Transcription
L	362	3.35	Replication, recombination and repair
B	42	0.30	Chromatin structure and dynamics
D	80	0.70	Cell cycle control, cell division, chromosome partitioning
Y	2	0.01	Nuclear structure
V	70	0.60	Defense mechanisms
T	236	2.18	Signal transduction mechanisms
M	179	1.65	Cell wall/membrane biogenesis
N	3	0.02	Cell motility
Z	51	0.40	Cytoskeleton
W	0	0	Extracellular structures
U	90	0.80	Intracellular trafficking and secretion
O	311	2.87	Posttranslational modification, protein turnover, chaperones
C	437	4.04	Energy production and conversion
G	813	7.52	Carbohydrate transport and metabolism
E	910	8.42	Amino acid transport and metabolism
F	129	1.19	Nucleotide transport and metabolism
H	250	2.31	Coenzyme transport and metabolism
I	409	3.78	Lipid transport and metabolism
P	616	5.70	Inorganic ion transport and metabolism
Q	600	5.55	Secondary metabolites biosynthesis, transport and catabolism
R	1772	16.40	General function prediction only
S	195	1.80	Function unknown
-	3295	30.49	Not in COGs

## Insights from the genome sequence

The whole genome sequence analysis of *A. niger* ATCC 10864 revealed the presence of several genes actively involved in biofilm formation, carbohydrate metabolism, and secondary metabolite biosynthesis. Currently, studies on the molecular mechanism of biofilm formation in the genera *Aspergillus* are limited. A previous report noted that acid protease-encoding gene pepA, sporulation regulating transcription factor brlA, and the beta-1,3-exoglucanase gene exgA may have a probable role during biofilm formation in *A. oryzae* because those genes were found to be expressed only in solid-state fermentation (SSF) when compared with submerged fermentation (SF) [31]. ExgA might play a role in glucan utilization and a combination of poor nutrition and mycelial attachment to a hydrophobic solid surface appears to be an inducing factor for exgA expression [31]. Genes like NADH:flavin oxidoreductase, alcohol dehydrogenase, malate dehydrogenase [32], transcription activator complex GCN, secondary metabolism regulator LaeA and cytoplasmic membrane protein coding gene rodA [33] may also have a possible influence during biofilm formation in *A. fumigatus*. A significant number of primary metabolism genes involved in sulfur amino acid biosynthesis and regulated by GCN are upregulated in *C. albicans* biofilms which also leads to the production of S-adeno-sylmethionine (SAM), a precursor of polyamines. Activation of the genes for SAM biosynthesis might be related to the production of a quorum-sensing molecule associated with biofilm formation [33]. The rodA gene belonging to the hydrophobins family encodes a cysteine-containing polypeptide that is assembled into a regular array of rodlets on the surface of conidia to render the surface highly hydrophobic. RodA gene has been reported to be upregulated in *A. fumigatus* biofilms [32]. We have detected all the aforesaid genes in *A. niger* ATCC 10864, which may regulate the process of biofilm formation. Gene Ontology (GO) terms for the annotated genes of *A. niger* ATCC 10864 were placed into three broad categories: biological process (BP), molecular function (MF), and cellular components (CC). A pie chart in the additional file represented the distribution pattern of some top level GO terms for the three categories (Additional file 1). In BP category the highest represented GO term was transmembrane transport (5.21%) followed by carbohydrate metabolic processes (2.43%) and transcription (1.7%). In MF category the most abundant GO terms include zinc ion binding (9.93%), ATP binding (7.53%) and oxidoreductase activity (5.71%). Integral component of the membrane (13.91%), nucleus (13.41%) and cytosol (5.96%) were the most representative GO terms in CC category. A total of 709 putative CAZymes families which are actively involved in carbohydrate metabolism have been identified in this study (e-value less than 10^−05^ has only been considered) and they were categorized into six functional classes such as Glycoside Hydrolases (GHs) = 279, Carbohydrate Esterases (CEs) = 134, Glycosyltransferases (GTs) = 123, Auxiliary Activities (AAs) = 107, Polysaccharide lyases (PLs) = 13, and Carbohydrate-Binding Modules (CBMs) = 53. Other genes involved in cellulose metabolism (Endoglucanase A, Endo-beta-1,4-glucanase D), xylan metabolism (Beta-xylanase), pectin metabolism (Endopolygalacturonase) and galactose metabolism (Galactose-1-phosphate uridylyltransferase, UDP-glucose 4-epimerase, Galactose oxidase) have also been identified.

Secondary metabolites (SMs) are small bioactive molecules which provide a competitive advantage to the fungi producing them in various ways. They may improve nutrient availability (e.g., in the form of chelating agents such as siderophores), protect it against environmental stresses (e.g., pigments against UV irradiation), enhance its competitive interactions for nutrients with other microorganisms in ecological niches, decrease the fitness of their hosts, e.g., plants, animals, or humans, and act as a metabolic defense mechanism [34]. The scaffold sequences of *A. niger* ATCC 10864 were analyzed for secondary metabolite gene clusters using antiSMASH and a total of 71 gene clusters were detected among which polyketide synthases (PKSs = 21) and nonribosomal peptides synthases (NRPSs = 21) were found to be most abundant. Secondary metabolite pathway annotation of *A. niger* ATCC 10864 was predicted by KAAS server using genus *Aspergillus* as reference and we have identified several genes that are mainly involved in caffeine metabolism (urate oxidase, xanthine dehydrogenase), indole diterpene alkaloid biosynthesis (geranylgeranyl diphosphate transferase, FAD-dependent monooxygenase), aflatoxin biosynthesis (acetyl-CoA carboxylase, norsolorinic acid ketoreductase, versiconal hemiacetal acetate esterase), carbapenem biosynthesis (glutamate-5-semialdehyde dehydrogenase, glutamate 5-kinase), monobactam biosynthesis (aspartate kinase, aspartate-semialdehyde dehydrogenase, sulfate adenylyltransferase), penicillin and cephalosporin biosynthesis (isopenicillin-NN-acyltransferase).

**Fig. 3:**
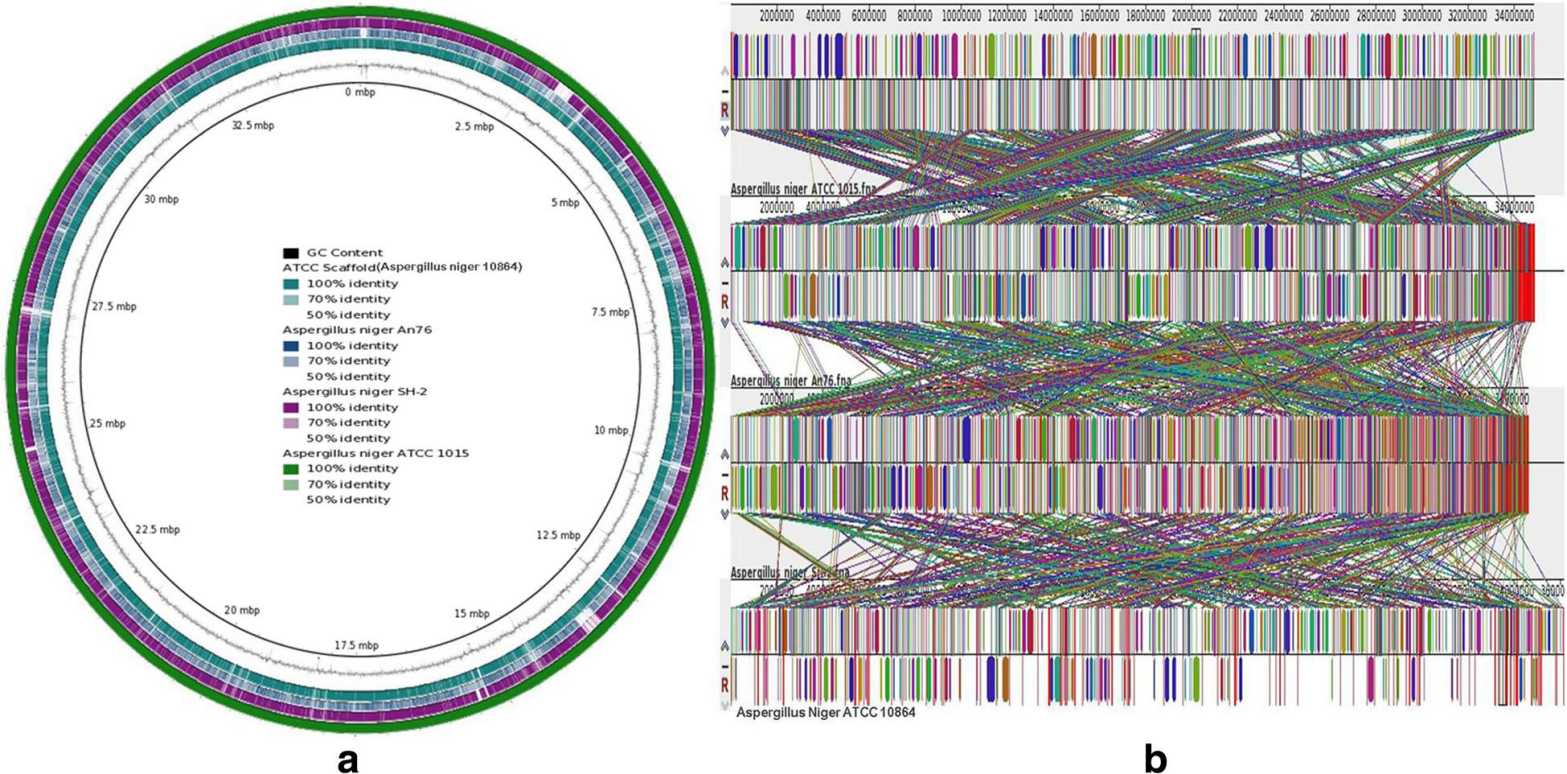
Whole genome comparison analysis of *A. niger* ATCC 10864 with other reported *A. niger* strains. **a** Comparison of *A. niger* ATCC 10864, *A. niger* An76, and *A. niger* SH-2 strains against reference *A. niger* ATCC 1015 strain (using BRIG tool). The outermost *dark green* circle represents the reference genome of *A. niger* ATCC 1015; the next *purple*, *blue* and *light green* circle represent *A. niger* SH-2, *A. niger* An76, and *A. niger* ATCC 10864 strains respectively. The *black* line lying in between the *A. niger* ATCC 10864 and genome-scale symbolize the GC content. **b** Comparison of *A. niger* ATCC 10864, *A. niger* An76, and *A. niger* SH-2 strains against reference *A. niger* ATCC 1015 strain (using Mauve tool). Colored block outlines are known as Locally Collinear Blocks and are connected by corresponding coloured lines. LCBs represent the regions of similarity among the genomes that are homologous and have not undergone any rearrangement. Blocks lying above the center *black* horizontal line are in forward orientation while blocks below the center line indicate regions that align in the reverse complement (*inverse*) orientation. Regions outside blocks (*white* regions) show no homology among the input genomes

A whole genome circular comparative map of *A. niger* 10,864 and other reported *A. niger* strains (ATCC 1015, An76, and SH-2) was generated using BRIG (Blast Ring Image Generator) v0.95 online tool [35]. All the scaffolds of *A. niger* ATCC 18064 were first stitched in a single scaffold and synteny map was constructed against the reference *A. niger* strain ATCC 1015. Each genome was represented by a different colour and the darkest areas in the circular genome displayed 100% sequence similarity with the reference genome, whereas the lightest (gray) areas showed 50% or less sequence similarity (Fig. 3a). From the BRIG analysis an overall of 85% similarity between the *A. niger* strain ATCC 10864 and *A. niger* strain ATCC 1015 is observed. Other two reference strains *A. niger* An76 and *A. niger* SH-2 shows approximately 81- 82% similarity against the *A. niger* ATCC 10864 strain (Fig. 3a). Multiple whole genome sequence alignment of the aforesaid strains was also performed using Mauve 2.3.1 [36] and *A. niger* 10,864 showed several non-homologous regions as compared to other *A. niger* strains (Fig. 3b).

## Conclusions

This is the first high-quality draft genome sequence report of an *A. niger* strain which can form a fungal biofilm. We selected this ATCC 10864 strain for genome sequencing not only for its unique biofilm forming character but also due to the fact that when it forms biofilm it can produce a higher amount of lignocellulolytic enzymes than free-living cultures. We expect that the high-quality genome report of *A. niger* ATCC 10864 strain will contribute to new insights about the role of fungal biofilms for higher biotechnologically important enzymes production, which could be highly beneficial in future for industrial purposes.

## Additional file

Additional file 1: Gene ontology pie chart of *A. niger* ATCC 10864. Distribution of Blast2GO annotations of putative genes from *A. niger* ATCC 10864. The charts show annotations for Biological Process, Molecular Function and Cellular Components. (PDF 346 kb)

## Abbreviations

CAZymes: Carbohydrate-Active Enzymes
COG: Cluster of Orthologous Groups
GRAS: Generally Recognized As Safe;
KAAS: KEGG Automatic Annotation Server
MIGS: Minimum information about a genome sequence
NAS: Non-traceable Author Statement
TAS: Traceable Author Statement
