# Supplementary material for: High-quality draft genome sequence of a biofilm forming lignocellulolytic Aspergillus niger strain ATCC 10864
Source: Stand Genomic Sci. 2017 Jul 17;12:37. doi: 10.1186/s40793-017-0254-2 (PMC5514509; doi:10.1186/s40793-017-0254-2)
Supplement: Additional file 1: — Gene ontology pie chart of A. niger ATCC 10864. Distribution of Blast2GO annotations of putative genes from A. niger ATCC 10864. The charts show annotations for Biological Process, Molecular Function and Cellular Components. (PDF 346 kb) [file 40793_2017_254_MOESM1_ESM.pdf]

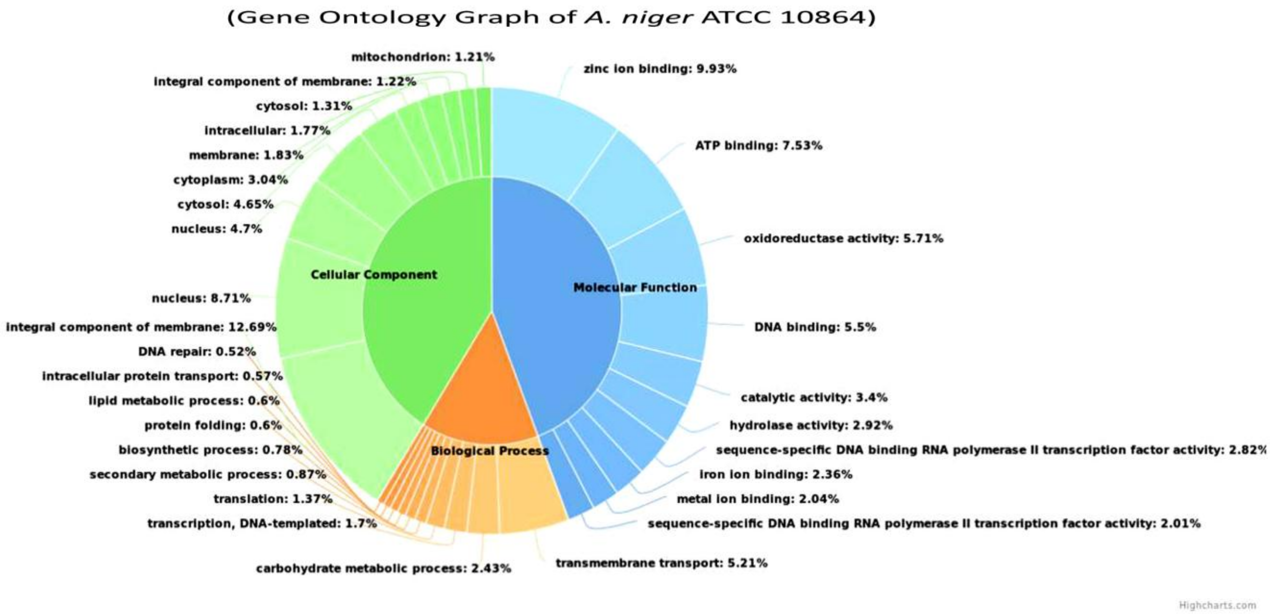

Figure. Distribution of Blast2GO annotations of putative genes from *A. niger* ATCC 10864. The charts show annotations for Biological Process, Molecular Function and Cellular Components.
